# Cortisol Awakening Response and Acute Stress Reactivity in First Nations People

**DOI:** 10.1038/srep41760

**Published:** 2017-01-31

**Authors:** Maximus Berger, Anthony Leicht, Angela Slatcher, Ann Katrin Kraeuter, Sarangan Ketheesan, Sarah Larkins, Zoltán Sarnyai

**Affiliations:** 1 Laboratory of Psychiatric Neuroscience, Australian Institute of Tropical Health and Medicine, 1 James Cook Drive, Townsville, 4811 QLD, Australia; 2 College of Public Health, Medical and Veterinary Sciences, 1 James Cook Drive, Townsville, 4811 QLD, Australia; 3 College of Healthcare Sciences, 1 James Cook Drive, Townsville, 4811 QLD, Australia; 4 Anton Breinl Research Centre for Health Systems Strengthening, AITHM, 1 James Cook Drive, Townsville, 4811 QLD, Australia; 5 College of Medicine and Dentistry, 1 James Cook Drive, Townsville, 4811 QLD, Australia

## Abstract

First Nations people globally have a higher incidence of mental disorders and non-communicable diseases. These health inequalities are partially attributed to a complex network of social and environmental factors which likely converge on chronic psychosocial stress. We hypothesized that alterations in stress processing and the regulation of the hypothalamic-pituitary-adrenal axis might underlie health disparities in First Nations people. We assessed the cortisol awakening response and the dynamic response to a laboratory induced psychosocial stress of young Indigenous tertiary students (n = 11, mean age 23.82 years) and non-Indigenous students (n = 11) matched for age and gender. Indigenous participants had a blunted cortisol awakening response (27.40 (SD 35.00) vs. 95.24 (SD 55.23), *p* = 0.002), which was differentially associated with chronic experience of stress in Indigenous (r = −0.641, *p* = 0.046) and non-Indigenous (r = 0.652, *p* = 0.03) participants. The cortisol response to the laboratory induced psychosocial stress did not differ between groups. Self-reported racial discrimination was strongly associated with flattened cortisol response to stress (r = −0676, *p* = 0.022) and with heart rate variability (r = 0.654, *p* = 0.040). Our findings provide insight into potential biological factors underlying health discrepancies in ethnic minority groups.

Ethnic minority groups are often confronted with social challenges that can affect their mental health. Migration is among the best-established risk factors for poor mental health^1^,^2^ and this risk persists well into the second generation^3^, indicating that it is not just the experience of migration that drives this increased risk but rather ethnic minority position^4^. Similarly, there is evidence that First Nations people who represent ethnic minorities in their own country have poorer health relative to the mainstream population^5^. There is mounting evidence that First Nations people globally are affected by worse mental health relative to the mainstream population, including Aboriginal Canadians^6^,^7^, Maori in New Zealand^8^ and Aboriginal and Torres Strait Islander (respectfully hereafter Indigenous) people in Australia, who are more than twice as likely to be hospitalised due to a mental disorder^9^. It is estimated that by closing the health gap in Australia, 5600 disability adjusted life years (DALYs) could be saved each year^10^. Despite substantial improvements in some areas such as infant mortality over the last decades, persisting health inequalities particularly in mental health remain a major source of disadvantage to First Nations people globally^5^.

Social determinants are thought to contribute substantially to the health inequalities affecting First Nations people. Social determinants include education and employment, culturally appropriate access to health care and prevention, adequate housing conditions, and freedom from racial discrimination^11^. In a broader context they also include control over life circumstances, empowerment and social inclusion^12^. Indigenous peoples and ethnic minority groups more generally continue to face a complex set of interacting social factors that likely converge on stress^13^ and may together drive a substantial proportion of the social gradient in health. Indeed, population-based studies show that Indigenous groups experience more stressful life events relative to the general population^14^,^15^. The notion that unequal exposure to stressful social and environmental factors shapes the ways in which the brain processes them has been highlighted as a potential pathway from social disadvantage to health inequalities^16^. Indeed, altered processing of social and environmental stimuli has been demonstrated in experimental designs^17^ and is thought to underlie health disparities in psychiatric epidemiology^18^.

Stress plays a major role in the development of a number of somatic and mental disorders and is a mediator of the effect of socioeconomic status on health^19^. Stress triggers the activation of the hypothalamic-pituitary-adrenal (HPA) axis, resulting in the production of glucocorticoids in the adrenal cortex^20^. These powerful hormones bind to glucocorticoid receptors (GR), which are expressed throughout the brain and body. Initially, this helps the body to react to acute stress and initiates a ‘fight or flight’ response. Chronically altered cortisol concentrations contribute to pathological processes termed ‘allostatic overload’, resulting in disease conditions such as metabolic syndrome, type-II diabetes, increased susceptibility to addictions and mental disorders, anxiety and depression, as well as cognitive impairment^21^,^22^. The concept of allostasis refers to chronic adaptation to changing demands of the HPA axis, the autonomic nervous system, immune system and metabolic function^23^. These mechanisms may be highly relevant to First Nation status associated risk as both exposure to stress^24^ and adverse health outcomes^5^ are well documented. Allostatic indices (e.g. cortisol) that are both biomarkers (that is, they are quantifiable) and mediators (as they exert effects on target tissues) of the effects of chronic stress exposure may serve as useful indices of longer-term health risks. However, such changes in neuroendocrine and autonomic function are poorly understood in First Nations people.

Therefore, the aim of this study was to examine the effect of chronic stress and discrimination on altered basal activity of the HPA-axis and the body’s multisystem response to social stress. Firstly, we hypothesised that Indigenous people would exhibit different circadian cortisol profiles relative to non-Indigenous people, specifically a heightened or attenuated cortisol awakening response (CAR). We further hypothesised that this would be explained by chronic stress and discrimination. Secondly, we hypothesised that the acute stress response of Indigenous people, measured with the Trier Social Stress Test (TSST), would be similarly altered (heightened or attenuated) and associated with the level of stress and discrimination experienced.

## Results

Of the 26 Indigenous and 26 non-Indigenous participants, 11 participants in each group completed all assessments, returned satisfactory data and were matched by age and gender. The dropout rate was 34.6% (9 of 26 subjects initially included). Four participants had missing data or returned empty saliva collection devices. One participant was excluded due to a pituitary adenoma, one participant was excluded from the analysis because of outliers (>3 SD above group mean) in two variables that appeared biologically implausible. Participants who did not complete the study were younger in the non-Indigenous group but older in the Indigenous group, and had higher somatic and anxiety scores in the Hopkins Symptom Checklist (HSCL). No difference in self-reported stress was found between participants who did and who did not complete the study. Indigenous participants reported significantly lower socio-economic status (SES) compared to non-Indigenous participants and we thus included SES as a covariate. No significant differences were found in any of the other socio-demographic variables (all *p* > 0.05, Table 1). Similarly, no statistically significant differences between Indigenous and non-indigenous students were found in scores for chronic stress, depressive symptoms, anxiety, adverse childhood events and number of negative life events in the past year (all *p* > 0.05, Table 2). Scores for perceived interpersonal racism in the previous year were high and 92.9% in the Indigenous group reported at least one event. Scores for internalised racism and systemic racism were similarly high (Table 2).

### Circadian Cortisol Excretion

**Figure 1:**
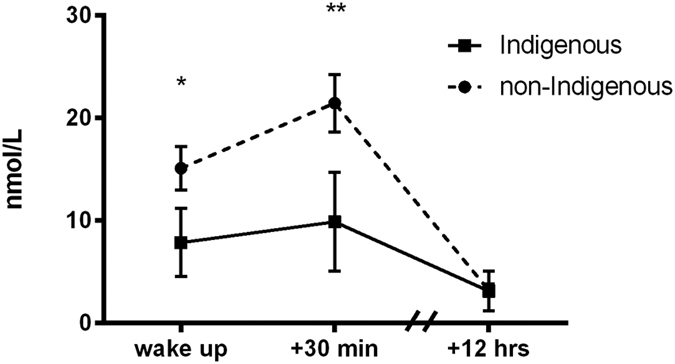
Circadian cortisol profile in Indigenous and non-Indigenous participants. Diurnal cortisol profiles of Indigenous and non-Indigenous participants. We collected three saliva samples across three days to assess diurnal cortisol profiles and found lower morning cortisol and lower cortisol 30 minutes after awakening. *p < 0.05, **p < 0.01.

The circadian cortisol pattern was significantly different between Indigenous and non-Indigenous participants (Fig. 1). Indigenous participants had significantly lower total cortisol output throughout the day (area under the curve (AUC) with respect to ground (AUCg); Fig. 1 and Table 2) and showed a significantly flatter CAR compared to non-Indigenous (AUC with respect to increase (AUCi); Fig. 1 and Table 2). Subsequent pairwise comparison revealed that Indigenous participants had significantly lower wake up cortisol and post-wake up cortisol compared to non-Indigenous participants. In contrast to this, evening cortisol was not significantly different between groups.

### Associations of the Circadian Cortisol Profile with Psychometric Measures

In non-Indigenous participants, chronic stress positively correlated with CAR (r = 0.728, *p* = 0.011), indicating that higher chronic stress was associated with a higher CAR (Table 3). Internalising was also associated with CAR (r = 0.623, *p* = 0.041). SES showed a negative correlation with CAR in non-Indigenous participants (r = −0.674, *p* = 0.023), indicating that lower SES was associated with a higher CAR.

In contrast to this, the opposite relationship between chronic experience of stress and CAR was observed in Indigenous participants. Chronic stress negatively correlated with CAR (r = −0.641, *p* = 0.046) (Table 3). Associations of circadian cortisol profile with depression, anxiety, somatisation and internalising and SES were not significant in Indigenous participants.

### Acute Psychosocial Stress

**Figure 2:**
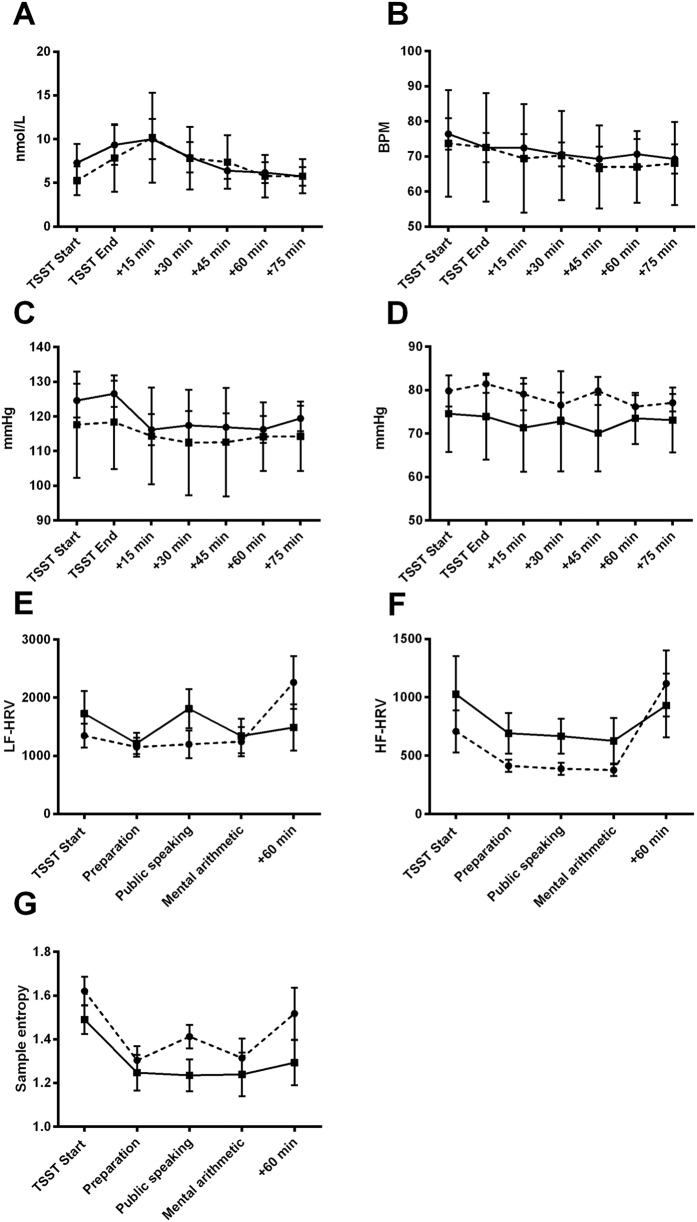
Neuroendocrine and autonomic response to the Trier Social Stress Test (TSST) in Indigenous and non-Indigenous participants. No significant between-group differences were observed in (**A**) cortisol, (**B**) heart rate, (**C**) systolic blood pressure, (**D**) diastolic blood pressure, (**E**) low-frequency heart rate variability, (**F**) high frequency heart rate variability or (**G**) sample entropy. Error bars are SEM.

The repeated measures ANOVA demonstrated a significant time effect for cortisol (F_(2.672)_ = 7.864, *p* < 0.001) (Fig. 2A), heart rate (HR) (F_(3.890)_ = 5.707, *p* < 0.001) (Fig. 2B) and systolic blood pressure (BP) (F_(3.931)_ = 4.850, *p* = 0.002) (Fig. 2C) in response to the TSST, indicating that the laboratory psychosocial stress paradigm induced a biological stress response. However, controlling for SES, no significant time x group interactions were found for HR (F_(3.657)_ = 0.214, *p* = 0.918), systolic BP (F_(3.873)_ = 0.771, *p* = 0.544) or cortisol concentration (F_(2.512)_ = 1.761, *p* = 0.177).

Both groups responded to the TSST with changes in heart rate variability (HRV), including decreases in low frequency (LF) HRV (F_(2.235)_ = 4.357, *p* = 0.016) (Fig. 2E), high frequency (HF) HRV (F_(2.392)_ = 6.738, *p* = 0.002) (Fig. 2F) and non-linear measures (sample entropy) (F_(2.604)_ = 5.846, *p* = 0.003) (Fig. 2G). However, no significant time x group interactions were detected.

### Associations of the Neuroendocrine and Autonomous Stress Response with Psychometric Measures

In Indigenous participants, the cortisol recovery from stress was associated with internalising (r = −0.775, *p* = 0.005) and the cortisol response to stress was associated with internalized racism (r = −0676, *p* = 0.022) (Table 4). Furthermore, significant associations were observed between social and emotional wellbeing (SEWB) and low HRV (Sample Entropy) during stress (r = −0.788, *p* = 0.012), depressive symptoms and low sample entropy during stress (r = −0.708, *p* = 0.033) and internalized racism and high sample entropy (r = 0.654, *p* = 0.040) in Indigenous participants only. In contrast to this, chronic perceived stress (r = 0.780, *p* = 0.005), internalising (r = 0.782, *p* = 0.004) and depressive symptoms (r = 0.809, *p* = 0.003) showed positive associations with HRV (Sample Entropy) in non-Indigenous participants (Table 4).

### Associations of Circadian Cortisol with Heart Rate Variability

In non-Indigenous participants, sample entropy during peak stress correlated significantly with CAR (r = 0.777, *p* < 0.005), but no such association was found in Indigenous participants. We observed, however, an association of LF/HF-ratio after recovery (+1 hour after the stress) with evening cortisol (r = −0.738, *p* = 0.037) in Indigenous participants, which was not found in non-Indigenous participants.

## Discussion

In the present study we tested the hypotheses that (i) First Nations groups at elevated risk for adverse health outcomes have alterations in basal HPA-axis regulation and the body’s multisystem response to social stress, (ii) and that such alterations are explained by unequal exposure to social and environmental factors. We detected significantly lower diurnal cortisol concentrations in Indigenous participants by characterising the circadian profile of the HPA-axis. Our approach of using three saliva samples per day across three different days further allowed us to characterise the morning increase in cortisol concentration and demonstrated a flattened CAR in Indigenous participants, characterised by the lack of increase within the first 30 minutes after waking up. The circadian profile found in non-Indigenous Australians in our study on the other hand is overall very similar to cortisol profiles found in other samples of young and healthy individuals^25^,^26^. This cortisol profile is characterised by a high CAR (approximately 50% increase), high 30 minutes post awakening levels and low evening cortisol. To the best of our knowledge, our study is the first one that studied trait and state markers of HPA-axis function in First Nations people and our observations are broadly consistent with previous research finding flattened diurnal rhythms in ethnic minority groups^25^.

Contrary to our hypothesis, no significant differences in cortisol excretion were found in response to acute psychosocial stress. This is consistent with two recent studies on HPA-axis stress reactivity in ethnic minority groups, which found differentially altered neural social stress processing but no difference in the peripheral cortisol response to stress^17^,^27^. Taken together with the basal differences between Indigenous and non-Indigenous participants in the current study, these results suggest different patterns of basal HPA axis function between Indigenous and non-Indigenous participants.

The blunted CAR for Indigenous participants found in this study may have significant implications for long-term health outcomes. Flattening of diurnal cortisol rhythms is observed in patients with common and severe mental disorders including depression^28^ and psychotic disorders^29^. Importantly, several social and environmental risk factors for neuropsychiatric disorders are associated with flattened CAR, including urban upbringing^30^ and childhood adversity^31^, which both raise the possibility that blunting of the CAR might indicate a risk for poor mental health. One longitudinal study found that flat CAR predicted mental health problems three years later^32^. The CAR is considered to be a necessary prerequisite to prepare the body for upcoming challenges of the day^33^. This hypothesis has been empirically validated^34^ and it has been demonstrated that a higher CAR is associated with better coping and less distress during the same day. In turn, a blunted CAR is associated with greater stress appraisal and more distress, indicating a relationship between trait markers of HPA-axis function and stress appraisal^35^. The mechanisms mediating the increased risk for illness later in life associated with blunting of the CAR remain mostly unknown. Besides associations with a pro-inflammatory state^36^, flat CAR is related to reductions in brain regions involved in stress processing including grey-matter volume reductions in the perigenual anterior cingulate cortex (pACC) and increased functional coupling between the pACC and the hypothalamus^37^. This directly links CAR to neural stress processing.

An important question raised by our data is whether the HPA-axis dysregulations found here are heritable, given the ubiquitous exposure to severe trauma of Indigenous Australian populations throughout the last two centuries. Insight into the heritability of HPA-axis abnormalities comes from twin studies that showed high heritability of CAR (0.56)^38^. This suggests that developmental, environmental and genetic factors substantially influence HPA axis function. In addition to these studies focusing purely on the heritability of neuroendocrine markers, the concept of trans-generational transmission of trauma is important here and may help to explain some of the findings. Trans-generational transmission of trauma is the vertical transmission of vulnerability for post-traumatic stress disorder (PTSD) from a trauma-affected individual onto the next generation. This hypothesis was formed in response to the observation that children of Holocaust survivors carry an increased risk for PTSD, despite not having been exposed to severe traumatic events^39^. Importantly, a core feature of this increased risk is hypocortisolism^40^. Further investigations revealed that these HPA-axis alterations are associated with the same epigenetic modifications in mothers and their children^41^,^42^. While the focus of these trans-generational effects on HPA-axis functions was PTSD, similar effects as a consequence of exposure to persistent stressors seem plausible. In fact, a trans generational effect of racism on health has been proposed^43^, but the role of the HPA-axis in this particular context remains unexplored. We did not observe statistically significant differences in exposure to childhood adversity or chronic perceived stress in our study; however, we cannot rule out that cumulative stressful experiences between childhood and adulthood that were not covered by our instruments or exposure to traumatic events in previous generations as described above impact on the HPA-axis. Considering the converging evidence on heritability of HPA-axis regulation and the trans-generational heritability of trauma, this offers a potential explanation for our findings.

Contrary to previous studies^9^, we did not observe higher levels of chronic perceived stress in Indigenous participants in our study. This may be due to the fact that we recruited study participants from a university setting, which has been found to be particularly stressful. Alternatively, we assessed chronic perceived stress only in the previous 30 days, which may not have captured earlier differential stress exposure. Interestingly, however, chronic perceived stress was differentially associated with CAR in Indigenous and non-Indigenous participants in our study, such that high chronic stress predicted a high CAR in non-Indigenous participants but a flat CAR in Indigenous participants. While self-reported stress levels were not different between the two groups, this may explain some of the differences in CAR and may indicate that HPA-axis regulation is differentially altered. It has to be noted though that we assessed chronic stress only in the last 30 days along with early childhood adversity, which does not allow assumptions about cumulative exposure to chronic stress. Converging evidence suggests that chronic exposure to social stress affects stress processing and HPA-axis regulation with relevance for psychiatric risk through genomic and non-genomic effects^44^,^45^,^46^. A previous meta-analysis of human studies investigating the effects of stress on HPA-axis regulation found that recent work stress and general life stress were associated with a higher CAR only in males^47^, which cannot explain our between-group differences as our subjects were matched for gender. We therefore suggest that chronic social stress may be responsible for the alterations in CAR observed in our study.

We did not observe significant differences in HRV between the two groups after adjusting our analysis for SES. However, the association of non-linear measures of HRV (sample entropy) during stress with depression scores and the association of HF-HRV with internalising in Indigenous participants suggests a potential link between affective symptoms and the autonomic response to stress. Chronic stress was only associated with higher non-linear measures during stress and recovery in non-Indigenous participants but no association was found in Indigenous participants. Decreased frequency domain measures^48^ and time domain measures^49^ of HRV have been reported in association with chronic stress and cumulative adversity, and decreased HRV was also found to be associated with racial discrimination^50^. Additionally, brain areas that play key roles in emotional regulation and anxiety are also involved in the control of HRV^51^. In light of the role of HRV in predicting cardiovascular morbidity, these findings seem highly relevant and warrant further investigation on HRV in vulnerable populations.

Previous studies consistently reported associations of low SES, particularly in early life, with elevated cortisol excretion^52^, a mechanism that was proposed to explain the relationship between low SES and high incidence of chronic diseases. As self-reported SES was not balanced between the two groups in our study it might therefore offer an alternative explanation for our findings. Therefore, we adjusted all between-group analyses of biological variables for SES. Other potential confounders including sleep duration, smoking and alcohol consumption were not significantly different between the groups.

Several limitations should be taken into account when interpreting these findings. Firstly, we recruited a sample of university students, a population that is not representative of all Indigenous people. This may limit the generalizability of our data; however, we argue that if such alterations in neuroendocrine and autonomic systems are observed in a high achieving group of young students, then one would expect to see more drastic effects in older or more disadvantaged groups. Secondly, our small sample size limits the analysis of psychometric and social variables in explaining HPA-axis regulation and stress response. Several variables were correlated (r > 0.3) but did not reach statistical significance, indicating that our study was not powered to detect smaller associations. Finally, a substantial percentage of the participants in both groups returned only some of their saliva samples, or empty collection devices. The reasons for this remain unknown but could be related to higher stress in those who did not adhere to the sampling protocol. To test this we explored differences between participants who adhered to the sampling protocol and those who did not and found no difference in self-reported stress. Expert consensus guidelines for assessment of CAR^53^ were published after our study was carried out and while our study complies with several important aspects of these guidelines (assessment of CAR across several days, using a sampling diary, instructing participants about specific requirements for saliva sample collections such to refrain from brushing their teeth prior to collecting a saliva sample), the guidelines also recommend the use of monitored sampling strategies such as electronic collection devices to objectively verify adherence to the sampling protocol. Strengths of the study include the assessment of a broad range of neuroendocrine and autonomic markers in a First Nations group while taking into account a variety of potential confounders including SES, sleep duration, smoking and alcohol consumption. Secondly, while most previous research on mechanisms linking ethnic minority position to adverse health outcomes focused on migrant populations in Europe, our study focused on Indigenous Australian people, and we can thus eliminate the potentially confounding effects of migration.

The altered HPA-axis function in Indigenous people in the present study is potentially relevant in terms of adverse health consequences^22^. While it has become clear that alterations in HPA-axis function and autonomic imbalance are associated with and predict adverse health outcomes, the findings of this study may have significant implications for the additional risk associated with Indigenous status underlying the health gap that affects First Nations people world-wide. Although these findings need replication in larger and broader groups, they provide insight into one mechanism (chronic dysregulation of the HPA-axis) of how exposure to psychosocial stress and social risk factors might translate into the high susceptibility to chronic disease that is seen in Indigenous communities.

## Method

### Participants

We recruited 26 students from James Cook University (JCU), Queensland, Australia who self-identified as Indigenous Australian using pin board posters on the university campus and print media advertisements, as well as at recruitment events organised specifically for this project. Twenty-six students who identified as non-Indigenous Australians were recruited as controls. Respondents were considered for inclusion if they were domestic students at JCU, identified either as Indigenous or as non-Indigenous Australians and did not fulfil any of the exclusion criteria. Exclusion criteria for both groups were prior participation in a stress experiment, any medical condition affecting the HPA axis, use of corticosteroids, bodybuilding and lack of capacity to consent. Control participants were carefully matched for age (+/− 2 years) and gender for comparison to a participant in the Indigenous group. All procedures contributing to this work were conducted in accordance with the ethical standards of the relevant national and institutional committees on human experimentation and with the Helsinki Declaration of 1975, as revised in 2008, i.e. the NHMRC Guidelines for Ethical Conduct in Aboriginal and Torres Strait Islander Health Research, and were approved by the Human Ethics Research Committee of JCU (approval number H5529). All study participants provided written informed consent.

### Demographic Measures and Psychological Assessments

Participants were asked to report subjective socioeconomic status on the McArthur ladder^54^. Subjective socioeconomic status was given preference over traditional measures of socioeconomic status (i.e. income, education, employment) as these would most likely not be relevant for students. Participants further reported work (hours per week), alcohol consumption, smoking and current medication. Stress exposure was assessed with the Perceived Stress Scale (PSS; Crohnbach’s α = 0.78)^55^ and the Kessler-6-Distress Questionnaire (K6; Crohnbach’s α = 0.89)^56^, which has been used in Indigenous populations previously. The Negative Life Events Scale was used to quantify negative life events and is validated for Indigenous people (Crohnbach’s α = 0.80)^57^. Childhood adversity was assessed with the Maltreatment and Abuse Expose Scale (MAES)^58^. We measured depression and anxiety with the HSCL (Crohnbach’s α = 0.86)^59^ as these are known to affect the stress response. Indigenous participants were asked to complete the Measure of Indigenous Racism Experience (MIRE; Crohnbach’s α = 0.83)^60^ to assess perceived racism and the Strong Souls Questionnaire (Crohnbach’s α = 0.70)^61^ to assess SEWB, a culturally appropriate measure of mental wellbeing among Indigenous people^62^. The latter two scales have been designed specifically for Indigenous Australian people.

### Laboratory Measures

Endogenous cortisol levels were analysed from saliva and the CAR, a longitudinally stable cortisol readout that is associated with vulnerability for mental disorders including mood disorders^28^ and psychotic disorders^29^,^63^, was computed as recommended by expert consensus guidelines^53^. CAR reflects the physiological increase in cortisol secretion within the first 30 minutes after waking up and was calculated as per Pruessner *et al*.^64^. Nine collection devices (Salivette^®^, Salimetrics™, California, USA) were handed out to each participant on the first study day and participants were instructed to collect three samples per day (at awakening, 30 minutes after awakening and at 9 pm) on three different days of the same week (Monday, Wednesday, Friday of the same week). A logbook was handed out to document the saliva collection. Participants were instructed not to eat or drink anything except water for 2 hours prior to collecting a sample and to store saliva samples below 4 °C. The samples were returned on the day when the acute stress experiment was conducted and the swab storage tubes were stored at −80 °C. Salivary cortisol was measured using an enzyme-linked immunosorbent assay (ELISA) kit (Salimetrics™, California, USA) with a detection limit of 0.007 μg/dL.

### Acute Laboratory Stress

We used the TSST to induce a biological stress response in participants. The TSST is a standardised laboratory stress procedure and reliably induces a stress response in humans. The procedure consists of a 20-minute anticipation period, followed by a 15-minute stress period and a 75-minute recovery period. Stress is induced with a public speaking task, during which participants are asked to give a talk in front of a panel of two researchers in a role-play scenario. This first task is followed by a mental arithmetic task of five minutes.

HR and BP during the TSST were measured immediately before and after the stress and at 15-minute intervals during the recovery period using an automated blood pressure monitor (Omron HEM-7130, Omron Healthcare, Hoofddorp, Netherlands). Seven saliva samples were collected during the procedure at 15-minute intervals using the Salivette^®^ collection tubes (Salimetrics™, California, USA). HR was measured continuously throughout the procedure using a HR monitor (Polar RS800CX, Polar Electrics Ltd., Kempele, Finland) with inter-beat intervals exported for later (HRV) analysis using dedicated software (Kubios v2.2, Department of Applied Physics, University of Eastern Finland, Kuopio, Finland) as previously described^65^. HRV was assessed in 5 minute blocks prior to the stress, during preparation and during each task of the TSST, and 60 minutes after the TSST.

### Statistical Analysis

For CAR and other diurnal cortisol variables, cortisol levels across the three days were averaged and mean values were used for the analysis. Mean values were computed for HR, BP, HRV and cortisol in response to the TSST. AUCg and AUCi were calculated as recommended by Pruessner *et al*.^64^ to calculate overall cortisol output and CAR respectively. Repeated-measures analyses of variance (ANOVA) and co-variance (ANCOVA) were conducted to determine within group and between group differences. Variables that were not balanced between the two groups were used as covariates. Normality of the data was ascertained with the Kolmogorov-Smirnoff test. T-tests and χ^2^ tests were calculated to assess differences in demographic variables and psychometric scores between the two groups. Likert scales were used in the analysis of psychometric variables rather than categorical cut-off values to allow correlational analyses. Pearson coefficients were computed to correlate continuous variables.

## Additional Information

**How to cite this article**: Berger, M. *et al*. Cortisol Awakening Response and Acute Stress Reactivity in First Nations People. *Sci. Rep.* **7**, 41760; doi: 10.1038/srep41760 (2017).

**Publisher's note:** Springer Nature remains neutral with regard to jurisdictional claims in published maps and institutional affiliations.

## Figures and Tables

**Table 1:** Demographic characteristics of Indigenous and non-Indigenous participants.

	Indigenous (n = 11)		Non-Indigenous (n = 11)		
	N (%)	M (SD)	N (%)	M (SD)	p-value
Age		23.82 (3.52)		23.18 (4.69)	0.422
Gender					1.000
male	3 (27.3%)		3 (27.3%)		
female	8 (72.7%)		8 (72.7%)		
Work					0.611
21–75 hrs/fortnight	2 (18.2%)		0 (0%)		
1–20 hrs/fortnight	3 (27.3%)		4 (40.0%)		
Not working	6 (54.5%)		6 (60.0%)		
Sleep					
Sleep duration		8:50:56 hrs		7:18:44 hrs	0.735
Wake-up time		7:31:48 am		6:48:11 am	0.187
Tobacco	1 (9.1%)		0 (0%)		1.000
Alcohol					0.676
>3 days/week	1 (9.1%)		2 (18.2%)		
≤3 days/week	8 (72.7%)		7 (63.6%)		
never	2 (18.2%)		2 (18.2%)		
Hormonal contraception	2 (25.0%)		2 (25.0%)		1.000
Antidepressants	2 (18.2%)		1 (9.1%)		1.000
SES		4.73 (1.35)		6.09 (1.04)	0.015

M = Mean, SD = Standard deviation, SES = Socio-economic status.

**Table 2:** Psychological measures, diurnal cortisol and cortisol stress response.

	Indigenous (n = 11)	Non-Indigenous (n = 11)			
	M (SD)	M (SD)	Test	Effect size (Cohen’s d)	p-value
Chronic stress (PSS)	15.27 (3.85)	17.09 (5.22)	T = 0.929	−0.40	0.364
Chronic stress (K6)	11.09 (2.43)	12.36 (3.20)	T = 1.105	−0.45	0.306
Depression	17.09 (5.49)	16.55 (2.66)	T = 0.841	0.13	0.410
Anxiety	9.36 (2.94)	8.46 (2.77)	T = 0.746	0.32	0.464
Negative life events	3.00 (1.95)	1.91 (2.63)	T = 1.107	0.47	0.282
Childhood adversity	33.40 (15.45)	25.13 (17.31)	T = 0.288	0.50	0.402
SEWB	12.80 (8.56)	na			
Depression	5.83 (2.92)	na			
Anxiety	1.78 (3.77)	na			
Suicide risk	0.28 (0.75)	na			
Resilience^1^	4.94 (3.80)	na			
Interpersonal racism	7.91 (7.08)	na			
Internalised racism	3.73 (1.10)	na			
Diurnal cortisol (AUCg) (nmol/h/L)	74.37 (38.53)	157.24 (64.77)	F = 11.893	−1.55	0.003^a^
CAR (AUCi) (nmol/min/L)	27.40 (35.00)	95.24 (55.23)	F = 13.553	−1.47	0.002^a^
Wake-up cortisol (nmol/L)	8.10 (3.23)	15.12 (7.11)	F = 6.991	−1.27	0.016^a^
+30 min cortisol (nmol/L)	9.89 (4.82)	21.47 (9.29)	F = 11.520	−1.56	0.003^a^
Evening cortisol (nmol/L)	3.14 (1.93)	3.21 (2.01)	F = 0.343	−0.04	0.566^a^
TSST cortisol stress response (nmol/L)	3.95 (5.02)	5.23 (4.94)	F = 0.195	−0.26	0.664^a^
TSST cortisol stress recovery (nmol/L)	6.02 (5.55)	4.72 (4.14)	F = 2.581	0.26	0.125^a^

PSS = Perceived Stress Scale, K6 = Kessler Distress Questionnaire, HSCL = Hopkins Symptoms Checklist, NLES = Negative Life Evens Scale, SWEB = social and emotional wellbeing, SSQ = Strong Souls Questionnaire, Measure of Indigenous Racism Experience; SSQ and MIRE were completed by Indigenous participants only. ^1^The resilience sub-scale of the SSQ is reverse-scored and a higher score therefore represents lower SEWB. ^2^Cortisol response to stress refers to the increase in cortisol from the lowest cortisol level observed before the Trier Social Stress Test to the highest cortisol level observed during or after the stressor. ^3^Cortisol recovery from stress refers to the decrease in cortisol from the highest cortisol level during the Trier Social Stress Test to the lowest cortisol level observed after cessation of the stressor. ^a^Adjusted for socioeconomic status.

**Table 3:** Pearson correlations between cortisol awakening response, stress response to and recovery from the Trier Social Stress Test.

	Cortisol awakening response				Cortisol stress response^a^				Cortisol stress recovery^b^			
	Indigenous		Non-Indigenous		Indigenous		Non-Indigenous		Indigenous		Non-Indigenous	
	R	*p*	R	*p*	R	*p*	R	*p*	R	*p*	R	*p*
Chronic stress (PSS)	−0.288	0.42	0.728	0.011	0.118	0.729	−0.576	0.064	0	0.999	−0.491	0.125
Chronic stress (K6)	−0.641	0.046	0.652	0.03	−0.037	0.915	−0.116	0.735	−0.15	0.661	0.081	0.813
SES	0.073	0.84	−0.674	0.023	−0.158	0.642	0.275	0.413	0.506	0.112	0.275	0.414
Internalising	−0.064	0.861	0.623	0.041	−0.449	0.166	−0.104	0.76	−0.775	0.005	0.129	0.705
Depression	−0.17	0.639	0.433	0.184	−0.162	0.634	−0.273	0.417	−0.551	0.079	−0.035	0.917
Anxiety	0.001	0.997	0.479	0.136	−0.297	0.376	−0.202	0.551	−0.583	0.06	−0.108	0.753
Negative Life Events	−0.001	0.998	0.233	0.491	−0.186	0.583	−0.252	0.455	−0.312	0.35	−0.289	0.389
SEWB	0.136	0.707			−0.112	0.757			−0.274	0.444		
Depression	0.022	0.952			−0.182	0.614			−0.521	0.122		
Anxiety	0.068	0.851			−0.103	0.778			0.029	0.936		
Suicide risk	0.147	0.686			−0.061	0.866			0.169	0.64		
Resilience	0.192	0.595			0.045	0.902			−0.223	0.536		
Interpersonal racism	−0.325	0.359			0.033	0.924			0.419	0.199		
Internalized racism	−0.433	0.212			−0.676	0.022			−0.107	0.755		

^a^Difference between baseline and peak-stress cortisol, ^b^Difference between peak-stress and recovery (+60 min) cortisol, Correlations were not calculated for childhood adversity due to n = 5 in one group.

**Table 4:** Pearson correlations between high-frequency heart rate variability and sample entropy during the Trier Social Stress Test.

	HF-HRV stress				HF-HRV recovery				Sample entropy stress				Sample entropy recovery			
	Indigenous		Non-Indigenous		Indigenous		Non-Indigenous		Indigenous		Non-Indigenous		Indigenous		Non-Indigenous	
	R	*p*	R	*p*	R	*p*	R	*p*	R	*p*	R	*p*	R	*p*	R	*p*
Chronic stress (PSS)	0.024	0.947	0.371	0.262	0.087	0.810	0.067	0.844	0.028	0.938	0.780	0.005	−0.154	0.672	0.659	0.027
Chronic stress (K6)	0.043	0.905	0.117	0.733	0.168	0.642	−0.149	0.662	0.156	0.666	0.759	0.007	−0.463	0.178	0.531	0.093
SES	0.741	0.014	−0.054	0.874	0.726	0.017	0.106	0.757	0.159	0.660	−0.522	0.100	0.382	0.276	−0.256	0.447
Internalising	−0.576	0.082	0.318	0.341	−0.633	0.049	−0.06	0.862	−0.485	0.155	0.782	0.004	−0.436	0.208	0.253	0.453
Depression	−0.364	0.301	0.17	0.618	−0.269	0.452	−0.292	0.384	−0.372	0.29	0.809	0.003	−0.509	0.133	0.605	0.049
Anxiety	−0.621	0.055	0.225	0.505	−0.533	0.112	−0.151	0.657	−0.579	0.08	0.503	0.115	−0.055	0.881	0.046	0.893
Negative Life Events	−0.317	0.373	−0.169	0.619	−0.263	0.462	−0.374	0.257	−0.352	0.318	0.127	0.709	−0.019	0.959	−0.063	0.855
SEWB	−0.614	0.078			−0.465	0.207			−0.788	0.012			0.004	0.993		
Depression	−0.666	0.050			−0.435	0.242			−0.708	0.033			−0.236	0.541		
Anxiety	−0.392	0.297			−0.236	0.540			−0.501	0.170			0.209	0.590		
Suicide risk	−0.246	0.524			−0.131	0.736			−0.485	0.186			0.153	0.695		
Resilience	−0.259	0.501			−0.347	0.361			−0.454	0.220			−0.072	0.854		
Interpersonal racism	0.357	0.311			0.587	0.074			−0.139	0.300			0.467	0.174		
Internalized racism	0.566	0.088			0.340	0.337			0.654	0.040			0.028	0.940		

HF-HRV = high frequency heart rate variability; correlations were not calculated for childhood adversity due to n = 5 in one group.
